# Mesorectal Lymph Node Metastases as Index Lesion in ^68^Ga-PSMA-PET/CT Imaging for Recurrent Prostate Cancer

**DOI:** 10.3389/fsurg.2021.637134

**Published:** 2021-03-01

**Authors:** Conrad Leitsmann, Marianne Schmid, Carsten-Oliver Sahlmann, Lutz Trojan, Arne Strauss

**Affiliations:** ^1^Department of Urology, University Medical Center Goettingen, Goettingen, Germany; ^2^Department of Nuclear Medicine, University Medical Center Goettingen, Goettingen, Germany

**Keywords:** prostate cancer, metastases, mesorectal lymph node, PSMA, ^68^Ga-PSMA-PET/CT imaging

## Abstract

**Purpose:** Several studies have demonstrated an advantage of ^68^Ga-PSMA-PET/CT as staging modality for detection of prostate cancer (PCa) metastases. Data concerning metastatic manifestation and impact on PCa development of mesorectal lymph nodes (MLN) is limited. Our investigation describes MLN metastases as index lesion in ^68^Ga-PSMA PET/CT imaging for recurrent PCa.

**Methods:** Twelve PCa patients with biochemical recurrence (BCR) after primary therapy who prospectively underwent a baseline ^68^Ga-PSMA-PET/CT initially showed MLN metastases. Eight of these patients received a follow-up ^68^Ga-PSMA-PET/CT to evaluate treatment response and further evolution. Prostate-specific antigen (PSA)-levels, changes in PSMA-uptake of MLN metastases and further ^68^Ga-PSMA PET/CT findings were recorded.

**Results:** Median PSA at the first ^68^Ga-PSMA-PET/CT was 5.39 ng/ml. In all patients therapeutic management changed after the first ^68^Ga-PSMA-PET/CT. Androgen deprivation therapy (ADT) was initiated in seven of eight patients, one patient restarted initial ADT. Three patients additionally received salvage radiation therapy (sRT) including the prostatic lodge and docetaxel chemotherapy was started in one case. At follow-up, a decrease of PSA-level was detected in all patients (median 2.05 ng/ml) after median 10 months. In six of eight patients we observed a decrease or complete regress of PSMA-uptake in MLN in the follow-up ^68^Ga-PSMA-PET/CT.

**Conclusion:** MLN metastases detected by ^68^Ga-PSMA-PET/CT seem to be a relevant localization of tumor manifestation and may serve as index lesion in the treatment of recurrent PCa. Besides the known oncological benefits of ADT and sRT, in case of sole MLN metastases individualized therapy like salvage lymphadenectomy or RT with a defined radiation field could be options for these patients.

## Introduction

In Europe the most common cancer in male is prostate cancer (PCa) with growing incidence in the past two decades (1). Radical prostatectomy (RP) and radiation therapy (RT) are curative therapeutic options (2). Nonetheless, within 10 years after primary therapy up to 40% of patients develop biochemical recurrence (BCR) (3). Here, due to limited sensitivity and specificity conventional imaging methods, such as computed tomography (CT) and magnetic resonance imaging (MRI), might struggle to accurately determine the presence or absence of metastatic or recurrent PCa (4, 5).

The Type-II transmembrane protein prostate specific membrane antigen (PSMA) is overexpressed in almost all PCa cells (6). Luiting et al. demonstrated promising results for detecting PCa relapse by Gallium (^68^Ga)-labeled PSMA positron emission tomography/computed tomography (PET/CT) (^68^Ga-PSMA PET/CT) (7). Further studies have confirmed the advantage of ^68^Ga-PSMA PET/CT compared to conventional imaging as well as functional ^18^F-choline-based PET/CT for patients with BCR (5, 8–11). Increasingly discussed salvage treatment of recurrent PCa also demands exact staging (4, 10, 12, 13). Roach et al. prospectively investigated the value of ^68^Ga-PSMA PET/CT in the management of PCa (14). They found that ^68^Ga-PSMA PET/CT scans detected previously unsuspected disease and assumed a greater impact in patients with BCR.

PCa typically spreads to the proximal external iliac, the lower sacral vessel, the obturator, the upper sacral, the common iliacal and, at last, the paraaortic lymph nodes (15). A previous retrospective analysis by Hijazi et al. however showed PCa metastases in mesorectal lymph nodes (MLN) in 12 of 76 patients with BCR, which were detected by ^68^Ga-PSMA PET/CT (11). Current studies addressing this issue are limited. Previous reports either described MLN metastases of PCa occurring in sentinel lymph node scintigraphy or as a random result during anterior rectal resection in patients with rectal cancer (16–19). We are considering MLN as a relevant region for lymph node metastasis in patients with recurrent PCa and as an important therapeutic target. The rationale behind is to further improve PCa-outcomes. A recent analysis by Horn et al. mentioned the correlation of a single lesion on PSMA PET/CT and low PSA as favorable prognosticators following PSMA-targeted radioguided surgery (20).

The current study presents observations of MLN metastases as index lesion for recurrent PCa and describes the depiction of treatment changes in patients with BCR and confirmed MLN metastases. In this context we discuss therapeutic options such as the surgical challenge of MLN dissection and the definition of the radiation field.

## Methods

### Patients' Characteristics

Patients with BCR after RT or RP or primary androgen deprivation therapy (ADT) were included. Twelve patients with BCR according to the EAU guidelines showed solitary MLN metastases in a baseline ^68^Ga-PSMA PET/CT (21). These 12 patients derive from a cohort, which was previously reported by Hijazi et al. of 76 patients with BCR, which were detected by 68Ga-PSMA PET/CT (11). One patient died during follow-up and was excluded from further investigation. Three of 12 patients were not available for follow-up. A total of eight patients received a follow-up ^68^Ga-PSMA PET/CT.

For each patient initial TNM (2), initial Gleason-Score, initial PSA-value (iPSA), year and type of primary treatment, date of the baseline ^68^Ga-PSMA PET/CT, date of the follow-up ^68^Ga-PSMA PET/CT, PSA-value at the follow-up ^68^Ga-PSMA PET/CT, treatment since the baseline ^68^Ga-PSMA PET/CT, treatment (change) after the second ^68^Ga-PSMA PET/CT, oncological status and PSA-value at follow-up were available. The study was performed as an individual diagnostic pathway per patient in consensus with the patient and was approved by the local Ethics Committee of the University Medical Center Goettingen (approval June 7, 2015).

### ^68^Ga-PSMA-PET/CT Imaging

Baseline ^68^Ga-PSMA PET/CT was performed between November 2014 and June 2015. Eight patients underwent a follow-up ^68^Ga-PSMA PET/CT between February and May 2016 to measure changes in the PSMA-uptake of MLN metastases or other PCa metastases. The ^68^Ga-PSMA PET/CT was performed as previously described (11, 22). An experienced nuclear medicine physician analyzed the images. The maximal standard uptake volume (SUV_max_) 1 and 3 h post injection was recorded.

### Statistical Analysis

Descriptive statistics of variables focused on frequencies. Means and standard deviations, medians and interquartile ranges were reported. Covariates consisted of initial TNM, initial Gleason-Score, iPSA, year and type of primary treatment, date of the baseline ^68^Ga-PSMA PET/CT, date of the follow-up ^68^Ga-PSMA PET/CT, PSA-value at the follow-up ^68^Ga-PSMA PET/CT, treatment since the first ^68^Ga-PSMA PET/CT, treatment change since the follow-up ^68^Ga-PSMA PET/CT, oncological status and PSA-value at follow-up.

All analyses were performed using Statistical Package for the IBM (SPSS, Inc., Chicago, IL, version 25). All parameters were analyzed with Fisher exact test.

## Results

### Patients' Characteristics

Characteristics of the total cohort are displayed in Table 1. Median age was 74 years (range 66–81 years), median iPSA was 19.25 ng/ml (range 4.6–90 ng/ml). Eight of the 12 patients underwent RP with standardized lymph node dissection as primary therapy (21). Two patients received primary ADT, one received a prostate-hyperthermia therapy and one received percutaneous RT. Median PSA at the first ^68^Ga-PSMA PET/CT was 5.39 ng/ml (range 0.31–67.21 ng/ml). Baseline ^68^Ga-PSMA-PET/CT showed predominantly one MLN metastasis (only one patient had two lesions). Time interval between primary therapy and first ^68^Ga-PSMA PET/CT was median 36 month (range 9–231 months). Median FU of the total study period was 32 months (range 29–38 months).

**Table 1:** Patients' characteristics of the initial study group (*n* = 12).

**ID**	**Age**	**TNM**	**iPSA (ng/ml)**	**Gleason score**	**PSA at first ^**68**^Ga-PSMA-PET/CT (ng/ml)**	**Initial treatment (date)**
1	76	pT2c (only Biopsy)	90	9	67.21	AA 11/2014
2	74	pT4, pN0, L1, V0, Pn1, R0	8	9	2.57	RP 03/09
3	75	pT2c, pN1, L1, V0, Pn1, R0	32	8	20	AA 12/2014
4	79	pT2b, pN1, L0, V0, Pn0, R0	93	9	23	Hyperthermia and immunotherapy 03/2014
5	66	pT2c, pN0, L0, V0, Pn0, R0	23	7b	0.31	RP 10/2012
6	79	pT3a, pN0, L0, V0, Pn0, R0	9.56	7a	1.84	RP 04/2013
7	59	pT3a, pN0, L1, V1, Pn0, R0	15.5	9	0.6	RP 03/2014
8	77	pT3b, pN0, L1, V1, Pn0, R0	12	6	6.18	RP 04/1996
9	81	pT3b, pN1, L1, V1, Pn0, R0	90	9	10	RP 19/2014
10	78	pT3b, pN0, L1, V1, Pn0, R0	5.6	8	0.6	RP 02/2012
11	54	pT2b (only Biopsy)	35	7	13	RT 03/2010
12	74	pT2c, pN0, L0, V1, Pn1, R0	4.6	9	4.6	RP 06/2015

*AA, Antiandrogen therapy; iPSA, initial Prostate specific antigen-value; RP, Radical prostatectomy; RT, Radiation therapy*.

### Follow-Up

Follow-up data are shown in Table 2. Median time between the two ^68^Ga-PSMA-PET/CT imaging was 10 months (range 8–14 months). A decrease of PSA serum value was demonstrated in all patients (median decrease −2.02 ng/ml, range −0.3 to −66.9 ng/ml). In all patients therapeutic management changed after the diagnosis of MLN metastases in the baseline ^68^Ga-PSMA-PET/CT. ADT [Luteinizing hormone-releasing hormone (LHRH) receptor agonists and antagonists] was initiated in seven of eight patients. One patient restarted initial ADT. Three patients received additional salvage RT including the prostatic lodge and docetaxel chemotherapy was started in one case. Three of these eight patients demonstrated a PSA <0.001 at the second ^68^Ga-PSMA-PET/CT.

**Table 2:** Patient's follow up data (*n* = 8).

**ID**	**PSA value at 1. scan**	**PSA value at 2. scan**	**PSA-difference (ng/ml)**	**Time between scans (months)**	**Treatment after first ^**68**^Ga-PSMA-PET/CT**	**Treatment after second ^**68**^Ga-PSMA-PET/CT**	**Follow-up-time (months)**	**PSA (ng/ml)**
1	67.21	0.7	−66.51	14	ADT (Trenantone)	ADT (Trenantone)	24	0.38
2	2.57	0.08	−2.49	14	ADT (Trenantone)	ADT (Trenantone)	22	0.001
5	0.31	0.001	−0.309	13	ADT (Trenantone)	ADT (Trenantone)	21	0.001
6	1.84	0.001	−1.839	10	RT + ADT (Trenantone)	ADT (Trenantone)	20	0.001
7	0.6	0.001	−0.599	10	ADT (Bicalutamid)	ADT (Bicalutamid)	22	0.001
8	6.18	3.98	−2.2	9	ADT (Trenantone) + Docetaxel	Best supportive care	–	–
11	13	9.35	−3.65	8	RT + ADT (Trenantone)	Chance ADT (Abiraterone)	22	13.5 (CR)
12	2	0.1	−1.9	10	RT + ADT (Firmagone)	ADT (Firmagone)	19	0.001

*AA, Antiandrogen therapy; CR, castration resistance within the FU; PSA, Prostate specific antigen; RP, Radical prostatectomy; RT, Radiation therapy (follow-up-time = months between second scan/treatment change until last follow up)*.

Oncologic treatments after the follow-up ^68^Ga-PSMA-PET/CT are shown in Table 2. Six of eight patients continued ADT and no change of treatment was needed. These patients showed hormone-sensitive PCa. In one patient antiandrogen therapy was changed (LHRH agonist replaced by Abiraterone acetate) as the follow-up ^68^Ga-PSMA-PET/CT showed a regression of the MLN metastasis but revealed one new metastasis next to the left kidney vessel. This patient developed castration resistant disease. Because of a massive disease progression decision for best supportive care strategy was made for one patient. For this patient we could not evaluate the castration level in the follow up.

PSA-values at the last follow-up between November 2017 and March 2018 [follow-up time median 22 months (range 19–24 months)] measured below 0.001 ng/ml in five of these six patients.

### Comparison of ^68^Ga-PSMA-PET/CT Imaging

We summarized results of the baseline and follow-up ^68^Ga-PSMA-PET/CT in Table 3. Median SUV_max_ of the MLN 3 h post injection (p.i.) was 7.5 (range 3.2–13.8). Only one patient showed more than one MLN metastasis in the baseline scan.

**Table 3:** Comparison of ^68^Ga-PSMA-PET/CT imaging (*n* = 8).

**ID**	**Mesorectal lymph node count in first scan**	**SUV_**max**_ 3 h p.i**.	**^**68**^Ga-PSMA-PET/CT findings**
1	1	13.8	No PSMA-uptake
2	1	10.5	Reduction of SUV_max_ to 3.5
5	1	3.2	No PSMA-uptake
6	1	–	No PSMA-uptake
7	1	7.5	No PSMA-uptake
8	1	7.6	Increase of one MLN metastasis (SUV_max_ 8.8), new metastases of the liver (SUV_max_ 7.1), retroperitoneal (SUV_max_ 12.7), and mediastinal (SUV_max_ 8.1); local recurrence (SUV_max_ 5.8)
11	2	5.5	Reduction of two MLN metastases (SUV_max_ to 3.7); new metastasis next to the left kidney vessels (SUV_max_ of 7.8)
12	1	6.1	No PSMA-uptake

*Ga, Gallium; PSMA, Prostate specific membrane antigen; SUV, Standard uptake volume; p.i., post injection*.

**Figure 1:**
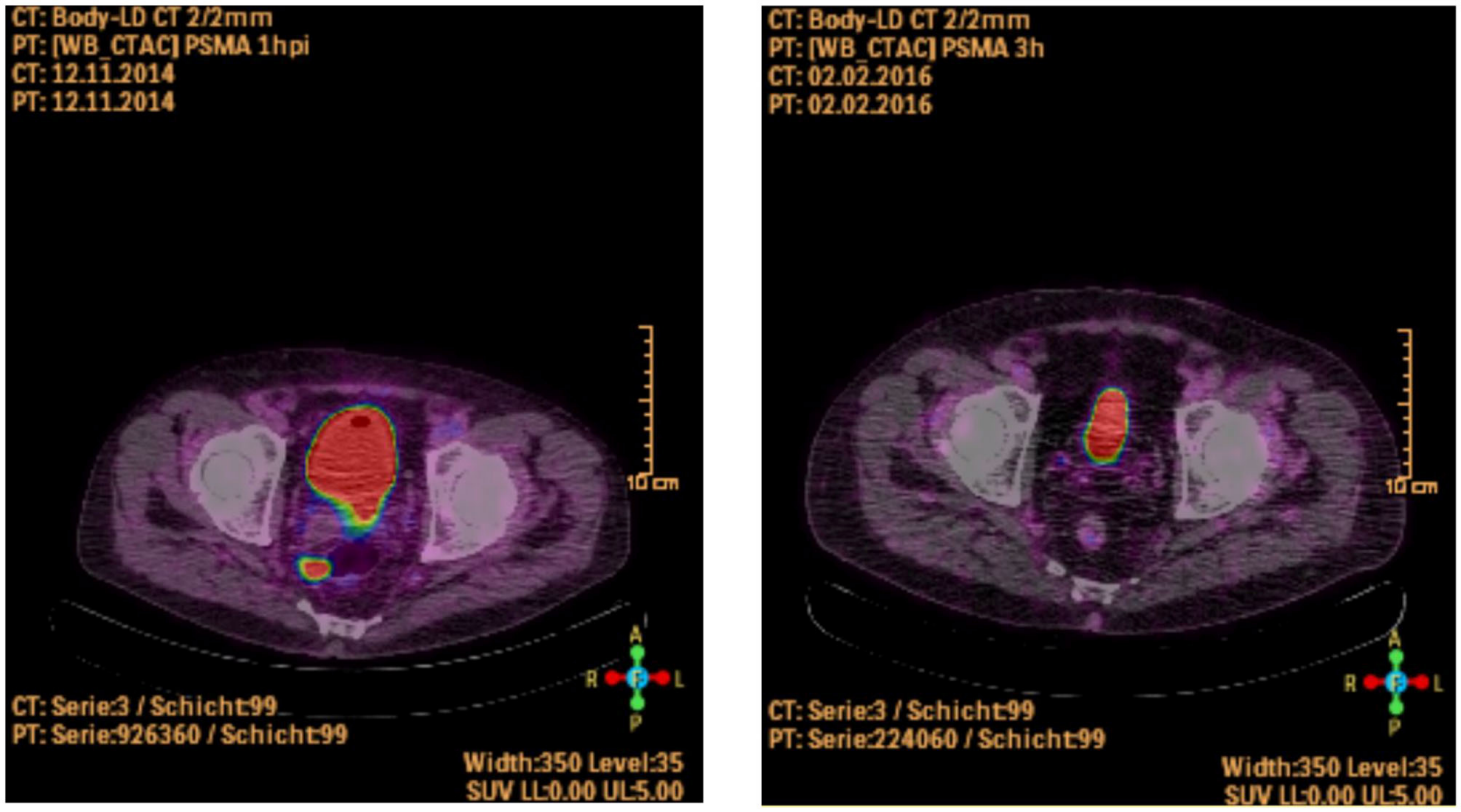
Patient with a singular MLN metastasis in the first ^68^Ga-PSMA-PET/CT (picture on the left side) and showing no more PSMA-uptake following ADT in the second scan after 13 months (picture on the right side, Patient ID 1).

In the follow-up scan five patients showed no more uptake of PSMA in ^68^Ga-PSMA-PET/CT imaging at all. An example is shown in Figure 1. The uptake declined in one patient from SUV_max_ 10.5–3.5 3 h p.i. One patient presented an increase of the one MLN metastasis from SUV_max_ 7.7–8.8 3 h p.i. and revealed new metastases in the liver (SUV_max_ 7.1 3 h p.i.), retroperitoneal (SUV_max_ 12.7 3 h p.i.), and mediastinal (SUV_max_ 8.1 3 h p.i.) as well as local tumor recurrence (SUV_max_ of 5.8 3 h p.i.). Another patient showed regression of the two MLN metastases (SUV_max_ 5.5–3.7 3 h p.i.), but the follow-up ^68^Ga-PSMA-PET/CT revealed one new metastasis next to the left kidney vessels (SUV_max_ of 7.8 3 h p.i.).

## Discussion

The aim of our study was to evaluate MLN metastases as “index lesion” in patients with recurrent PCa. Hijazi et al. showed solitary metastasis in MLN detected by ^68^Ga-PSMA-PET/CT in 12 of 76 patients (11). We could include eight of these 12 patients into a follow-up and present our observations. All of these patients started ADT with LHRH agonists or antagonists after the first scan. Two of these six patients were additionally treated with RT including pelvic lymph nodes and the prostatic bed. Six patients showed definitive benefit of secondary treatments and PSA-values decreased significantly (*p* < 0.01). ADT and RT resulted in complete regress or at least reduced PSMA-uptake of MLN metastases in six of eight patients in the follow-up ^68^Ga-PSMA-PET/CT. Additionally, PSA was <0.001 ng/ml in three of these patients at the second ^68^Ga-PSMA-PET/CT and in five at last follow-up. Usually, in PCa patients with PSA-values below 0.001 ng/ml after treatment further imaging would not be recommend. However, previous investigations report of superior imaging and detection of metastases with ^68^Ga-PSMA PET/CT (14). As our study intended to investigate its value in treatment monitoring, five patients with PSA-values below 0.001 ng/ml underwent a follow-up ^68^Ga-PSMA-PET/CT in this context. In these patients no additional metastases were found.

In the current literature comparable studies are rare. For a long time MNL have been never considered as a route of PCa spread. Murray et al. and Mourra et al. identified metastases of PCa in MLN in 4.5 and 9.4% of histologically examined lymph nodes in patients who underwent anterior rectal resection (16, 17). These studies demonstrated a relevant number of patients with MLN metastases of PCa. Additionally, Swanson et al. showed in an investigation of lymphatic drainage of PCa that PCa rarely metastasizes in MLN and that an assessment of nodal staging based on obturator lymph node dissection had an accuracy of 50% (15). A standard dissection of pelvic lymph nodes does not include the mesorectum region or the posterior pelvic subsite (PPS) (23).

Current data showed a promising opportunity using ^68^Ga-PSMA-PET/CT for assessment of lymph node metastases in PCa patients with BCR prior to salvage lymphadenectomy (24). These studies use this fact to investigate possible PSMA-guided surgery in these patients (25). A very recent review revealed PSMA-PET/CT as an indicator for metastasis targeted therapies in patients with recurrent PCa (26). So, it is most important to exactly detect and locate lymph node metastases before salvage treatment of PCa, as well as in recurrent PCa. This could affect the choice of treatment strategies like salvage lymphadenectomy or PSMA-guided surgery that has been proved to be successful concerning intraoperative detection and removal of metastatic lesions promises to improve prognosis of recurrent PCa-patients (27). However, patients with MLN metastases of PCa, like demonstrated in our study, are a small subgroup of patients which have unfavorable ratio of risk/benefit for a salvage lymph dissection because of the difficult surgical approach. Due to the difficulty of the surgical approach to MNL metastases three of eight patients of our study therefore underwent adjuvant RT. Two of these had no PSMA-uptake in the second scan and a significant decrease of PSA-values. Although our study could not prove an additional positive effect of adjuvant RT due to the small number of patients, this option may serve as possible treatment modality for patients with MLN metastases. Resent publications used ^68^Ga-PSMA-PET/CT for the planning of RT (28, 29). Schiller et al. and Habl et al. showed a significant influence of the higher detection rate of PCa lesions with ^68^Ga-PSMA-PET/CT imaging for radiation planning in recurrent PCa patients allowing individually personalized treatment compared to conventional CT or MRI staging. Of note, unless lymph node involvement is assured, the posterior pelvic subsite (PPS) below S3 or the mesorectal region is usually not comprised in the radiation filed of PCa (30).

Limitations of our study include the small number of patients, heterogenic baseline oncologic parameters like iPSA or Gleason and an incomplete follow-up of all 12 patients. Additionally, no comparison or control group was present. We focused on patients with mesorectal lymph node metastases to evaluate this index lesion and we did not investigate the total initial study group with a follow up PSMA-PET/CT. Reasons were the justification of exposure to radiation and the high rate of patients lost to follow up. Two patients seemed to have no benefit of the initiated treatment. One patient showed a new PSMA-positive lymph node next to a kidney vessel. A possible reason for this new metastasis might concern the irradiation area, which was focused on pelvic lymph nodes and the prostatic fossa and not on the aortocaval lymph node region. One patient presented a severe tumor progression in the follow-up ^68^Ga-PSMA-PET/CT. This patient had the longest time interval between the initial PCa therapy and the change of oncological treatment (9 years) and was one of the oldest patients in our cohort (77 years).

Further studies with larger number of patients need to be performed to evaluate the relevance of our findings. Future investigations should focus on the potential benefit of individual RT vs. (challenging) guided surgery vs. systemic treatments.

## Conclusions

Recent studies showed an optimized detection of metastasis in recurrent PCa by ^68^Ga-PSMA-PET/CT imaging. This investigation used MLN metastases as index lesion. Considering such a rare localization like the mesorectum reveals individualized treatment options for such patients and may lead to improved oncological outcomes.

## Data Availability Statement

The raw data supporting the conclusions of this article will be made available by the authors, without undue reservation.

## Ethics Statement

The studies involving human participants were reviewed and approved by Ethic Committee, University Medical Center Goettingen. The patients/participants provided their written informed consent to participate in this study.

## Author Contributions

CL, LT, and AS: protocol/project development, data collection and management, data analysis, and manuscript writing. MS: manuscript editing and data collection and management. C-OS: protocol/project development, data collection and management, and data analysis. All authors contributed to the article and approved the submitted version.

## Conflict of Interest

The authors declare that the research was conducted in the absence of any commercial or financial relationships that could be construed as a potential conflict of interest.
